# An Elementary Approximation of Dwell Time Algorithm for Ultra-Precision Computer-Controlled Optical Surfacing

**DOI:** 10.3390/mi12050471

**Published:** 2021-04-21

**Authors:** Yajun Wang, Yunfei Zhang, Renke Kang, Fang Ji

**Affiliations:** 1 School of Mechanical Engineering, Dalian University of Technology, Dalian 116023, China; jjswang@xjtu.edu.cn (Y.W.); kangrk@dlut.edu.cn (R.K.); 2 Institute of Machinery Manufacturing Technology, China Academy of Engineering Physics, Mianyang 621900, China; jfang2013@caep.cn

**Keywords:** ultra-precision machining, computer-controlled optical surfacing, dwell time algorithm, removal function, elementary approximation

## Abstract

The dwell time algorithm is one of the key technologies that determines the accuracy of a workpiece in the field of ultra-precision computer-controlled optical surfacing. Existing algorithms mainly consider meticulous mathematics theory and high convergence rates, making the computation process more uneven, and the flatness cannot be further improved. In this paper, a reasonable elementary approximation algorithm of dwell time is proposed on the basis of the theoretical requirement of a removal function in the subaperture polishing and single-peak rotational symmetry character of its practical distribution. Then, the algorithm is well discussed with theoretical analysis and numerical simulation in cases of one-dimension and two-dimensions. In contrast to conventional dwell time algorithms, this proposed algorithm transforms superposition and coupling features of the deconvolution problem into an elementary approximation issue of function value. Compared with the conventional methods, it has obvious advantages for improving calculation efficiency and flatness, and is of great significance for the efficient computation of large-aperture optical polishing. The flatness of φ150 mm and φ100 mm workpieces have achieved PVr_150_ = 0.028 λ and PVcr_100_ = 0.014 λ respectively.

## 1. Introduction

With the rapid increasing requirements for the fabrication of high-precision optical elements in modern optical systems, several advanced deterministic optical surfacing technologies have been developed over the past decades, such as ultra-precision computer controlled optical surfacing (CCOS), magnetorheological finishing (MRF), ion-beam figuring (IBF), bonnet polishing (BP) [1,2]. These achieve precision material removal on certain workpiece areas by accurately controlling the dwell time on the elaborately predesigned polishing path. Therefore, the dwell time algorithm is one of the key elements in modern advanced deterministic optical surfacing technologies. Among them, most of the removal functions have the characteristics of rotational symmetry, but how to use this feature to develop a high-efficiency, high-precision dwell time algorithm is the current research focus.

In deterministic optical finishing technologies, the amount of material removal can be expressed as discrete two-dimensional convolution operations of the dwell time and removal functions. The dwell time algorithm is used to solve the deconvolution process and to ensure that the calculated dwell time not only meets the performance of machine tools, but also has a high surface error convergence efficiency. Various dwell time algorithms have earlier been proposed, including the Fourier transform method, the numerical iteration method, the matrix equation method. Ronald Aspden et al. [3] studied the polar and rectangular coordinates of the process in CCOS, and discussed the variation of the removal function with the radius of the workpiece in the gyrosymmetric correction process. Jones et al. [4] proposed an iterative method for solving the dwell time function, studied the relationship between the flatness convergence efficiency and the removal function, and pointed out that only the symmetric central single-peak removal function could converge. The convergence accuracy of these methods is not high enough, and now they are seldom applied. Carnal et al. [5] introduced the linear equation method and solved the dwell time by adopting the LSQR method. Drueding et al. [6] proposed a series expansion solution. Waluschka et al. [7] presented a one-dimensional dwell time function algorithm for cylindrical workpieces based on a graphic method, and Shanbhag et al. [8] proposed an algorithm based on wavelet analysis. Zheng et al. [9] proposed a damped iterative method for solving the CCOS dwell time function. Zhou et al. [10] used the TSVD method to solve the linear equation model, which entailed further research. Wu et al. [11] proposed a solution based on discretized matrix equations using LSQR. Jiao et al. [12] and Jiang et al. [13] improved the traditional L–R algorithm. Taking into account the scanning path of the polishing tool and the tilt angle of the workpiece, Guo [14] proposed a dwell time algorithm to achieve rapid convergence of the accuracy of the optical mold. Pan et al. [15] proposed an improved dwell time calculation algorithm to optimize tool path planning in optical figuring. Li et al. [16] developed a positive dwell time algorithm with minimum equal extra material removal to consider the machine dynamics limitations. Li and Zhou [17] gave a solution algorithm of dwell time in a slope-based figuring model. Wang et al. [18] provided a quantitative study on the performances of dwell time algorithms in ion-beam figuring. Han et al. [19,20] proposed a Gaussian mixture model to model experimental removal functions and provided the dwell time algorithm according to the dynamic characteristics of the machine tool. These methods are mainly based on matrix equations, and the computational efficiency might be much lower especially for large-aperture optical elements, so the solution might not be smooth enough.

The existing dwell time algorithms are conducted mainly based on a meticulous mathematical theory and designed to pursue high convergence rate. Nonetheless, those methods do not adequately consider the distribution characteristics of the removal function and rarely incorporate the speed-smoothing issues that are closely related to convergence efficiency and machine tool motion implementation. Actually, the convergence rate of flatness is only between 1.1X and 1.3X [21] for most CNC machines; hence, it is unnecessary to pursue high convergence rates excessively.

In this paper, an elementary approximation method for solving the dwell time algorithm based on the symmetrical distribution of single-peak rotation of removal function is proposed. The proposed method has the characteristics of clear physical meaning and was verified by simulation and experiments. By using triangular approximation of the removal function, the initial surface shape is discretized reasonably and the approximate solution of dwell time is obtained. In this paper, the performance of the algorithm is verified through residence-time mathematical modeling, accuracy analysis, simulation and experimental research. The results showed that it performs well in the profile for smoothness and convergence efficiency.

## 2. Dwell Time Algorithm Model

### 2.1. Approximation Treatment of Removal Function

According to the measured residual error, the surface is polished to achieve a theoretical profile. To eliminate the residual error, the material removal function, generated by the polishing tool in a constant time (also called the removal function) [22,23,24], and the dwell time of the polishing tool should be known first. It is generally assumed that deterministic optical surfacing technology is a linear shift-invariant system, and the mathematical model of the convolution of the dwell time and removal functions being equal to the distribution of the removal amount is generally adopted, and this is given in Equation (1) [6]:

(1) Δh(x,y)=R(x,y)∗∗D(x,y)

where ∆*h(x, y)* is the distribution function of removal amount, *R(x, y)* is the removal function (also called influence function), and *D(x, y)* is the dwell time function.

**Figure 1:**
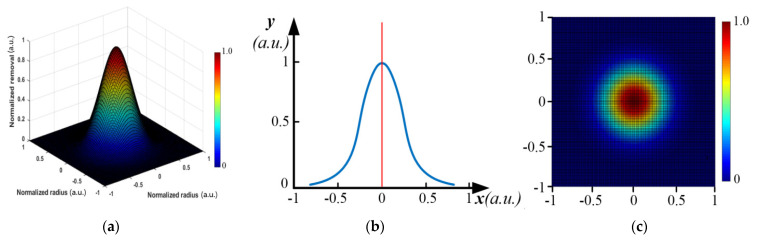
The basic distribution of removal function in some deterministic optical surfacing technologies: (**a**) Two-dimensional distribution of ideal Gaussian removal function, (**b**) One-dimensional distribution of CCOS removal function, (**c**) Kernel function distribution of ion beam figuring (IBF).

In some deterministic optical surfacing technologies, the removal function is distributed by rotational symmetry. Suppose an ideal removal function is a two-dimensional Gaussian distribution, as shown in Figure 1.

**Figure 2:**
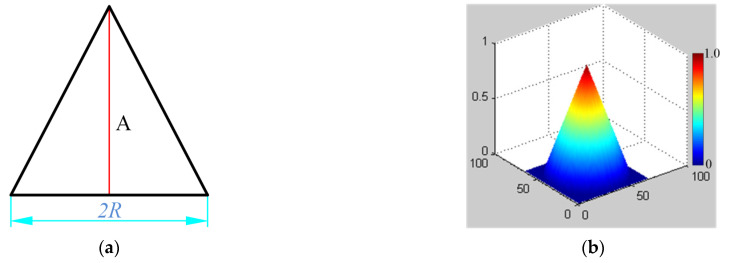
Approximate distribution of the removal function: (**a**) One-dimensional approximation, (**b**) Two-dimensional approximation.

According to the actual characteristics of its distribution [25], the removal function is approximated as shown in Figure 2.

In the one-dimensional case, the removal function can be represented by an isosceles triangle distribution with height *A* and bottom 2*R*, similar to the roof function in the one-dimensional finite element method. In the two-dimensional case, it is assumed that the kernel function can be represented by a conic distribution with a height of *A* and a bottom radius of *R*. This kind of approximation reflects the main distribution characteristics of the removal function and concentrates more than 80% of volume removal, which is completely acceptable in engineering.

### 2.2. Dwell Time Algorithm Model

#### 2.2.1. One-Dimensional Analysis

**Figure 3:**
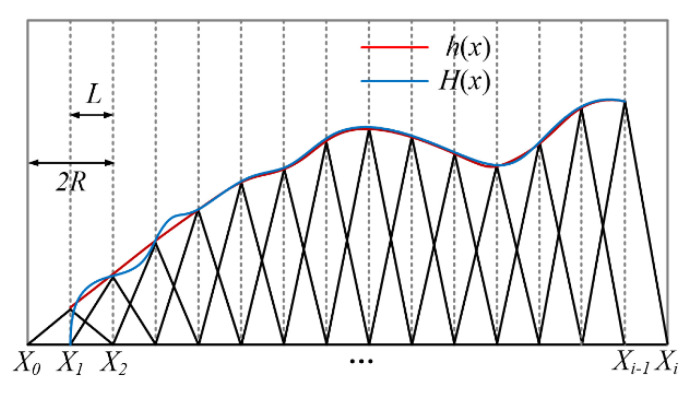
Elementary approximation solution for one dimension.

The principle of an elementary approximation for a one-dimensional deconvolution is shown in Figure 3. The blue curve represents the target removal amount distribution curve *H*(*x*), and the red curve is the actual removal amount distribution curve *h*(*x*). The standard removal function has a maximum width of 2*R* and a height of *A*. The discretization distance of the nodes is *L* = *R*. For each dwell node *X_i_*, the dwell time function is set to *D_i_* = *H*(*X*_i_)/*A*, which eliminates superposition coupling and is only a simple elementary algebraic operation. It shows that the algorithm has the same accuracy as the trapezoidal method of the one-dimensional definite integral problem, and its approximation residual is a second-order small quantity.

**Figure 4:**
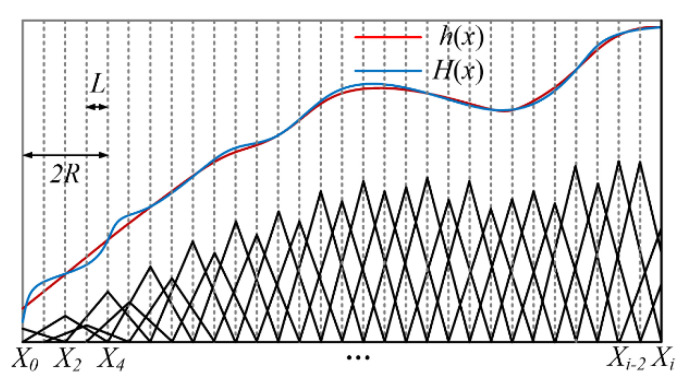
Mesh refinement principle for one dimension.

When *L* = *R*, the calculated residual error is already a second-order small quantity, but it can still be seen that the actual removal curve is not very smooth, which means that the smoothness of the optical processing surface is poor. When the discretization node is doubled and the spacing *L* = *R*/2, then *D_i_* = *H*(*X*_i_)/2*A*. The calculation principle of elementary geometric approximation for one-dimensional mesh refinement is shown in Figure 4.

Generally, let *L* = *R*/2*^n^*, and the distribution function of target removal amount is *H*(*x*) = 2*^n^*·*H*(*x*)/2*^n^*. The discretized node set {X_i_} is divided into 2*^n^* groups. The node spacing in each group is *L* = *R*, and the phase difference between each group of nodes is an integer multiple of *R*/2*^n^* in turn. Each set of nodes after partition is decoupled according to Figure 3, and then *D_i_* = *H*(*X_i_*)/2*^n^*/*A*. In this way, the actual processing curve gradually becomes smooth.

According to the above analysis, the basic criteria for the elementary approximation of one-dimensional deconvolution are as follows:

1. It is acceptable to use an isosceles triangle as an approximate expression of the removal function in engineering;
2. The discretization distance of the nodes should not be more than half of the width of the removal function; otherwise, the deconvolution calculation will lose the ability to approximate;
3. When the node spacing is doubled, the time weight of each node is reduced by half, so the total time remains basically unchanged. The dwell time of the subdivided nodes is not the interpolation between the original discrete nodes, but the redistribution of the dwell time. The physical meaning is that the total removal amount is constant, and the removal function is constant, so the total time is basically conserved;
4. The approximation residual of approximate solution is the same as that of definite integral trapezoid method, which is a second-order small quantity.

#### 2.2.2. Two-Dimensional Analysis

**Figure 5:**
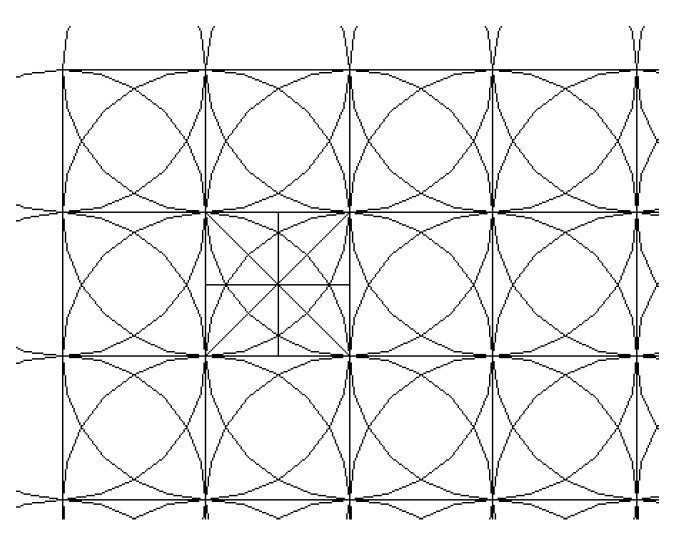
Mesh generation.

The orthogonal grid *M* is divided according to the spacing *R*. The coordinates of each grid node are (*x_i_*, *y_j_*), and the target removal amount on the node is H (*x_i_*, *y_j_*). The plane distribution is discussed first, as shown in Figure 5.

Let the removal function be a conic distribution and the center of the conic be the origin. The expression under the rectangular coordinate system is shown as follows:

(2) $$\left\{ \begin{array}{l} \varphi_{ij}\left(x,y\right)=\frac{A}{R}\left(R-\sqrt{{\left(x-x_{i}\right)}^{2}+{\left(y-y_{j}\right)}^{2}}\right),{\left(x-x_{i}\right)}^{2}+{\left(y-y_{j}\right)}^{2}\leq R^{2}\\ \varphi_{ij}\left(x,y\right)=0,{\left(x-x_{i}\right)}^{2}+{\left(y-y_{j}\right)}^{2}>R^{2} \end{array}\right.$$

Where *R* is the radius of the circular support region of the removal function, and *A* is the peak value of the removal function center. The expression in polar coordinate form is shown as follows:

(3) $$\left\{ \begin{array}{c} \varphi_{ij}\left(\rho ,\theta \right)=\frac{A}{R}\left(R-\sqrt{{\left(\rho \cos\theta -\rho_{ij}\cos\theta_{ij}\right)}^{2}+{\left(\rho \sin\theta -\rho_{ij}\sin\theta_{ij}\right)}^{2}}\right),\\ \sqrt{{\left(\rho \cos\theta -\rho_{ij}\cos\theta_{ij}\right)}^{2}+{\left(\rho \sin\theta -\rho_{ij}\sin\theta_{ij}\right)}^{2}}\leq R\\ \varphi_{ij}\left(\rho ,\theta \right)=0,\sqrt{{\left(\rho \cos\theta -\rho_{ij}\cos\theta_{ij}\right)}^{2}+{\left(\rho \sin\theta -\rho_{ij}\sin\theta_{ij}\right)}^{2}}>R \end{array}\right.$$

On the mesh, the removal function is simplified as follows:

(4) $$\left\{ \begin{array}{l} \varphi_{i}\left(x\right)=\frac{A}{R}\left(R-fabs(x-x_{i})\right),{\left(x-x_{i}\right)}^{2}\leq R^{2}\\ \varphi_{i}\left(x\right)=0,{\left(x-x_{i}\right)}^{2}>R^{2} \end{array}\right.$$

Or the following polar form:

(5) $$\left\{ \begin{array}{l} \varphi_{j}\left(y\right)=\frac{A}{R}\left(R-fabs(y-y_{j})\right),{\left(y-y_{j}\right)}^{2}\leq R^{2}\\ \varphi_{j}\left(y\right)=0,{\left(y-y_{j}\right)}^{2}>R^{2} \end{array}\right.$$

Where *fabs* represents the function of taking absolute value.

Suppose that each node (*x_i_*, *y_j_*) of the grid is superimposed with a removal function *φ_ij_* of Equation (2) above, which has the same weight. Then, for the interior of the region M, according to the symmetry, only the case of the middle region *R* × *R* needs to be considered. The edge of the whole grid area is special and will not be discussed here.

**Figure 6:**
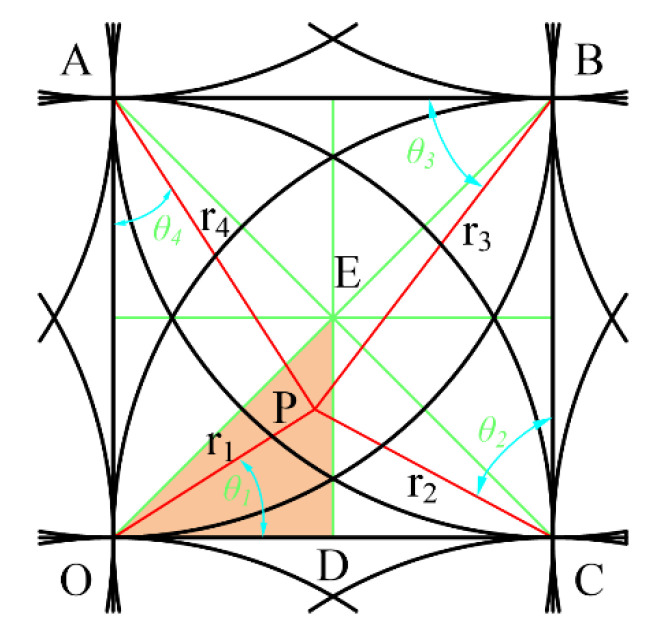
Superposition distribution map in grids.

For the grid area, it is customary to take the lower left corner of the grid as the origin O, so the center of the removal function is respectively located at the four corners of the grid, as shown in Figure 6. The green lines are auxiliary lines, the red lines are distances from each corner nodes, and P is an arbitrary point in the polishing area. Set the node number of O as (*i, j*), OP = *r_1_*, ∠POC = *θ*_1_, CP = *r*_2_, ∠PCB = *θ*_2_, BP = *r*_3_, ∠PBA = *θ*_3_, AP = *r*_4_, and ∠PAO = *θ*_4_ to give the following:

(6) $$\left\{ \begin{array}{l} r_{ij}=0,\theta_{ij}=0\\ r_{ij+1}=R,\theta_{ij+1}=0\\ r_{i+1j+1}=\sqrt{2}R,\theta_{i+1j+1}=\frac{\pi }{4}\\ r_{i+1j}=R,\theta_{i+1j}=\frac{\pi }{2} \end{array}\right.$$

According to Equation (3), there are the following:

(7) $$\left\{ \begin{array}{l} \varphi_{ij}\left(r_{1},\theta_{1}\right)=\frac{A}{R}\left(R-r_{1}\right)\\ \varphi_{ij+1}\left(r_{2},\theta_{2}\right)=\frac{A}{R}\left(R-r_{2}\right)\\ \varphi_{i+1j+1}\left(r_{3},\theta_{3}\right)=\frac{A}{R}\left(R-r_{3}\right)\\ \varphi_{i+1j}\left(r_{4},\theta_{4}\right)=\frac{A}{R}\left(R-r_{4}\right) \end{array}\right.$$

For a discrete mesh, due to the symmetry, only one-eighth of the triangular EOD area in the grid needs to be considered. This area can be further divided into four subareas: boundary line, M1, M2, and M3. For each subregion, only the value range of any point in the region under the function of each removal function (the maximum and minimum values) can be considered for evaluating the approximation ability of the approximate solution. The points where the maximum and minimum are located are the feature points in each square. According to the symmetry, these feature points must be obtained on the symmetry axis or the boundary of the square.

Similar to the one-dimensional case, let *L* = *R*/2*^n^*, and the distribution function of target removal amount is *H*(*x*) = 2*^n^*·*H*(*x*)/2*^n^*. The discretized node set (*x_i_*, *y_j_*) is divided into 2^n^ groups. The node spacing in each group is *L* = *R*, and the phase difference between each group of nodes is an integer multiple of *R*/2*^n^* in turn. Each set of nodes after partition is decoupled as above, and then there is *D_i_* = *H*(*x_i_*, *y_j_*)/(2*^n^A*). The basic criteria for the primary approximation of two-dimensional deconvolution are as follows:

(1) It is acceptable to use cone as an approximate expression of the removal function in engineering.
(2) The distance of node discretization should not be larger than the radius of the removal function support domain; otherwise, the deconvolution calculation based on this method will lose the ability to approximate.
(3) When the node spacing is doubled, the time weight of each node is reduced by half, so the total time remains basically unchanged.
(4) The approximation residual of the elementary geometric approximation method for two-dimensional deconvolution is completely acceptable compared with the actual polishing convergence rate.

### 2.3. Dwell Time Algorithm Analysis

#### 2.3.1. Split Line Value Analysis

**Figure 7:**
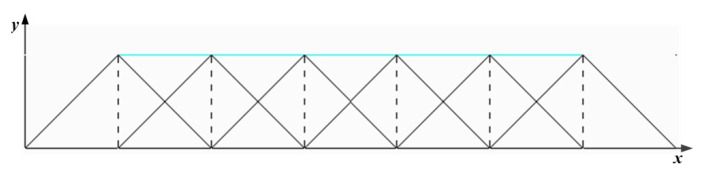
Schematic diagram of value taken on longitude and latitude line.

In the real polishing case, the workpiece surface is a two-dimensional planar. For each meridian segment or latitude segment, there are only two kernel functions. At this time, the superposition value of the removal function is always A, as shown in Figure 7.

For example, let *x_i_* + 1 > *x* > *x_i_*, *x_i_*_+ 1_ = *x_i_* + *R,* then the value on the grid is determined by Equation (8).

(8) $$f=\varphi_{i}\left(x\right)+\varphi_{i+1}\left(x\right)=\frac{A}{R}\left(R-(x-x_{i})\right)+\frac{A}{R}\left(R-(x_{i+1}-x)\right)=A$$

#### 2.3.2. Area M1 Value Analysis

**Figure 8:**
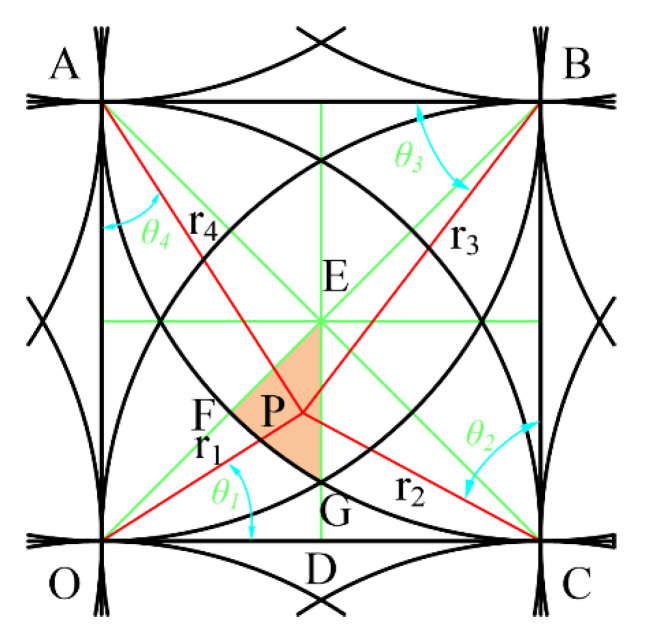
Four kernel function scopes.

The point P is located in the region M1 and is acted on by four removal functions as shown in Figure 8.

According to Equations (3), (6) and (7), at this time the value of any point in M1 is determined by Equation (9).

(9) $$f=\varphi_{ij}\left(r_{1},\theta_{1}\right)+\varphi_{ij+1}\left(r_{2},\theta_{2}\right)+\varphi_{i+1j+1}\left(r_{3},\theta_{3}\right)+\varphi_{i+1j}\left(r_{4},\theta_{4}\right)=\frac{A}{R}(4R-(r_{1}+r_{2}+r_{3}+r_{4}))$$

The relationships of each parameter are as follows:

(10) $$\begin{array}{l} r_{2}=\sqrt{{\left(r_{1}\cos\theta_{1}\right)}^{2}+{\left(R-r_{1}\cos\theta_{1}\right)}^{2}},\theta_{2}=Arcctg(\frac{r_{1}\sin\theta_{1}}{R-r_{1}\cos\theta_{1}})\\ r_{3}=\sqrt{{\left(r_{2}\cos\theta_{2}\right)}^{2}+{\left(R-r_{2}\cos\theta_{2}\right)}^{2}},\theta_{3}=Arcctg(\frac{r_{2}\sin\theta_{2}}{R-r_{2}\cos\theta_{2}})\\ r_{4}=\sqrt{{\left(r_{3}\cos\theta_{3}\right)}^{2}+{\left(R-r_{3}\cos\theta_{3}\right)}^{2}},\theta_{4}=Arcctg(\frac{r_{3}\sin\theta_{3}}{R-r_{3}\cos\theta_{3}})\\ (\sqrt{2}-1)R\leq r_{1}\leq \frac{\sqrt{2}}{2}R,\frac{5\pi }{36}\leq \theta_{1}\leq \frac{\pi }{4} \end{array}$$

According to the symmetry, when point P is located at the vertex G of EFG, that is, *r*_3_ = *r*_4_ = *R*, $r_{1}=r_{2}=\sqrt{2-\sqrt{3}}R$, making the sum of *r*_1_+*r*_2_+*r*_3_+*r*_4_ the maximum, then the minimum value of the function in this area is:

(11) $$f_{\min}=2(1-\sqrt{2-\sqrt{3}})A\approx 0.9647A$$

Since the sum of any two sides of a triangle is greater than the third side, it can be known that when P is located at the center of the square; that is, $r_{1}=r_{2}=r_{3}=r_{4}=\sqrt{2}R/2$, the sum of *r*_1_+*r*_2_+*r*_3_+*r*_4_, is a minimum, so the maximum value in this area is

(12) $$f_{\max}=4\frac{A}{R}(R-\frac{\sqrt{2}}{2}R)\approx 1.172A$$

#### 2.3.3. Area M2 Value Analysis

**Figure 9:**
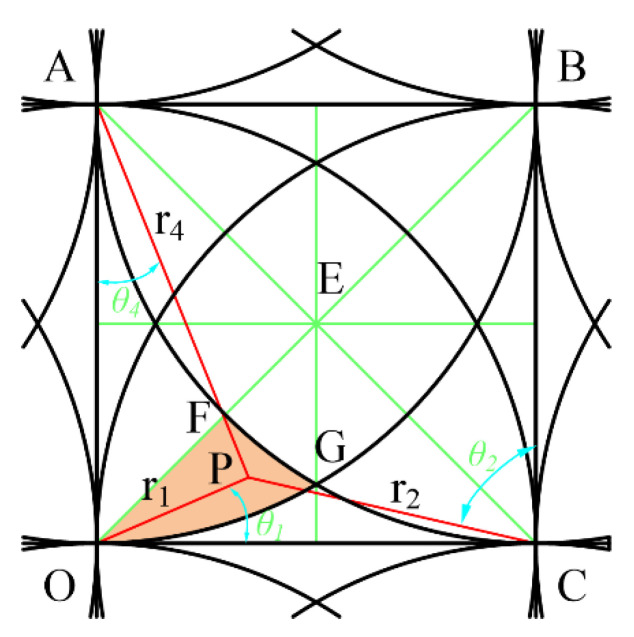
Three removal function scopes.

The point P is located in the region M2 and is acted on by three removal functions as shown in Figure 9.

According to Equation (3), (6) and (7), at this time, the value of any point in M2 is determined by Equation (13).

(13) $$f=\varphi_{ij}\left(r_{1},\theta_{1}\right)+\varphi_{ij+1}\left(r_{2},\theta_{2}\right)+\varphi_{i+1j}\left(r_{4},\theta_{4}\right)=\frac{A}{R}(3R-(r_{1}+r_{2}+r_{4}))$$

The relationships of each parameter are as follows:

(14) $$\begin{array}{l} r_{2}=\sqrt{{\left(r_{1}\cos\theta_{1}\right)}^{2}+{\left(R-r_{1}\cos\theta_{1}\right)}^{2}},\theta_{2}=Arcctg(\frac{r_{1}\sin\theta_{1}}{R-r_{1}\cos\theta_{1}})\\ r_{3}=\sqrt{{\left(r_{2}\cos\theta_{2}\right)}^{2}+{\left(R-r_{2}\cos\theta_{2}\right)}^{2}},\theta_{3}=Arcctg(\frac{r_{2}\sin\theta_{2}}{R-r_{2}\cos\theta_{2}})\\ r_{4}=\sqrt{{\left(r_{3}\cos\theta_{3}\right)}^{2}+{\left(R-r_{3}\cos\theta_{3}\right)}^{2}},\theta_{4}=Arcctg(\frac{r_{3}\sin\theta_{3}}{R-r_{3}\cos\theta_{3}})\\ 0\leq r_{1}\leq \sqrt{2-\sqrt{3}}R,0\leq \theta_{1}\leq \frac{\pi }{4} \end{array}$$

According to the symmetry, when point P is located at the vertex G of EFG, that is, *r*_4_ = *R*, $r_{1}=r_{2}=\sqrt{2-\sqrt{3}}R$, making the sum of *r*_1_+*r*_2_+*r*_4_ the maximum, then the minimum value of the function in this area is:

(15) $$f_{\min}=2(1-\sqrt{2-\sqrt{3}})A\approx 0.9647A$$

According to the symmetry, when point P is located at the vertex G of EFG, that is, *r*_1_ = 0, *r*_2_ = *r*_4_ = *R*, making the sum of *r*_1_ + *r*_2_ + *r*_4_ the minimum, then the maximum value in this area is:

(16) $$f_{\max}=A$$

#### 2.3.4. Area M3 Value Analysis

**Figure 10:**
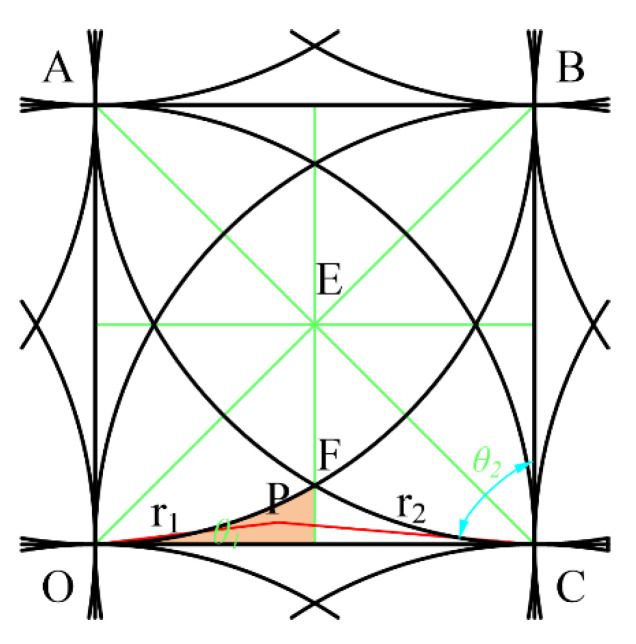
Two removal function scopes.

The point P is located in the region M2 and is acted by two removal functions as shown in Figure 10.

According to Equation (3), (6) and (7), at this time, the value of any point in M3 is determined by Equation (17).

(17) $$f=\varphi_{ij}\left(r_{1},\theta_{1}\right)+\varphi_{ij+1}\left(r_{2},\theta_{2}\right)=\frac{A}{R}\left(2R-(r_{1}+r_{2})\right)$$

The relationships of each parameter are as follows:

(18) $$\begin{array}{l} r_{2}=\sqrt{{\left(r_{1}\cos\theta_{1}\right)}^{2}+{\left(R-r_{1}\cos\theta_{1}\right)}^{2}},\theta_{2}=Arcctg(\frac{r_{1}\sin\theta_{1}}{R-r_{1}\cos\theta_{1}})\\ 0\leq r_{1}\leq \sqrt{2-\sqrt{3}}R,0\leq \theta_{1}\leq \frac{5\pi }{36} \end{array}$$

According to the geometric relationship and symmetry, when point P is located at the vertex G of a curved triangle, that is, $r_{1}=r_{2}=\sqrt{2-\sqrt{3}}R$, the sum of *r*_1_ + *r*_2_ is the maximum, then the following function obtains the minimum value:

(19) $$f_{\min}=2(1-\sqrt{2-\sqrt{3}})A\approx 0.9647A$$

When point P is located on the side of the curved triangle OD, that is, *r*_1_ + *r*_2_ = *R*, the sum of *r*_1_ + *r*_2_ is the smallest, and the function obtains the maximum value:

(20) $$f_{\max}=A$$

#### 2.3.5. Numerical analysis

**Figure 11:**
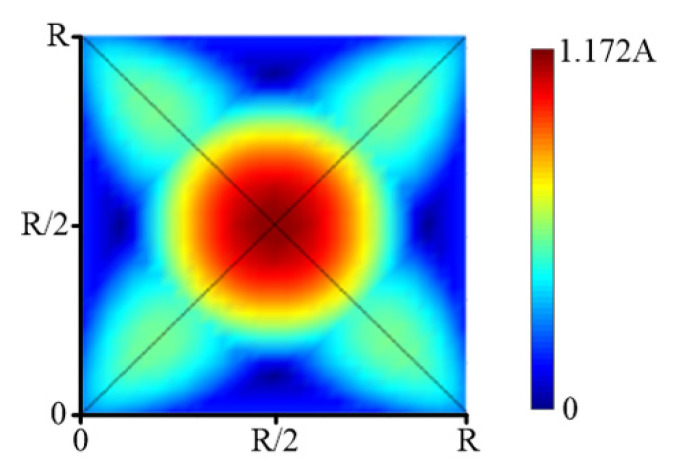
Simulation results of removal function superposition.

Figure 11 is a cloud picture of simulation calculation of an equal weight superposition distribution of removal function in the middle area. The distribution characteristics are consistent with the theoretical analysis.

According to the results of the above analysis, on the whole grid the maximum and minimum values of the weight superposition distribution of the removal function are as follows:

(21) $$\begin{array}{l} f_{\min}=2(1-\sqrt{2-\sqrt{3}})A\approx 0.9647A\\ f_{\max}=4(1-\frac{\sqrt{2}}{2})A\approx 1.172A \end{array}$$

This reflects the approximation ability of the conic distribution removal function to the plane, that is, the error level based on the elementary approximation method. The convergence accuracy of the calculation is much higher than the actual polishing convergence rate.

More generally, if each removal function is weighted according to the value of the surface distribution at the center, the conical distribution removal function can approximate the general surface better. That is to say, for any initial error distribution, the dwell time function the kernel of which is a cone distribution can be approximately determined by the weight of the kernel’s center. At this time, the higher deconvolution problem can be simplified to a basic function-value calculation problem.

## 3. Simulations

**Figure 12:**
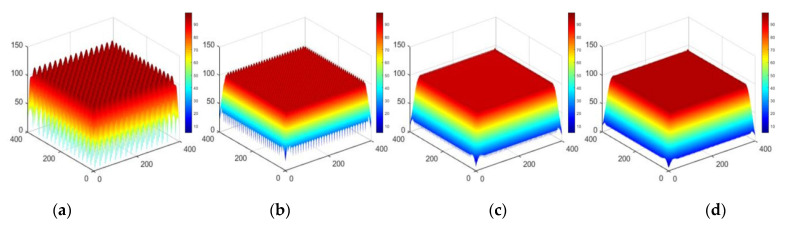
Approximation results for the distribution of plane removal amount (**a**) Grid spacing L = R, (**b**) Grid spacing L = R/2, (**c**) Grid spacing L = R/4, (**d**) Ideal plan distribution.

**Figure 13:**
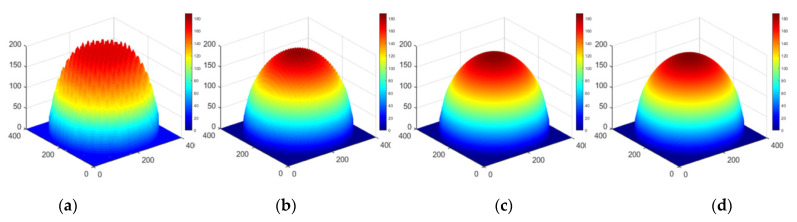
Approximation results for the distribution of spherical removal (**a**) Grid spacing *L* = *R*, (**b**) Grid spacing *L* = *R*/2, (**c**) Grid spacing *L* = *R*/4, (**d**) Ideal hemispheric distribution.

**Figure 14:**
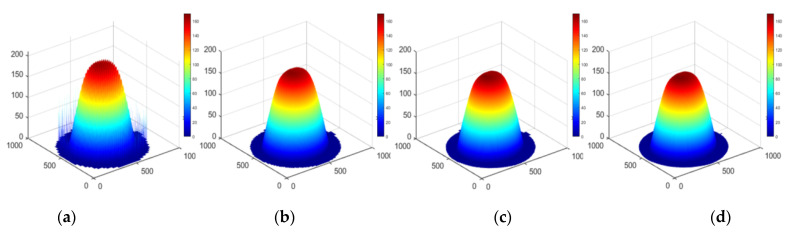
Approximation results for the distribution of arbitrary removal (**a**) Grid spacing *L* = *R*, (**b**) Grid spacing *L* = *R*/2, (**c**) Grid spacing *L* = *R*/4, (**d**) Original surface distribution.

The example of a two-dimensional deconvolution based on an elementary approximation is shown below. Three sets of simulations use the cone distribution removal function, and the removal amount distribution is a plane, sphere and arbitrary surface, respectively. The dwell time function is solved by the elementary approximation method proposed in this paper. The results are shown in Figure 12, Figure 13 and Figure 14.

Apparently, the numerical simulation results of a deconvolution calculation based on an elementary approximation are satisfactory.

## 4. Results and Discussion

### 4.1. Experiment Setup and the Parameters

**Figure 15:**
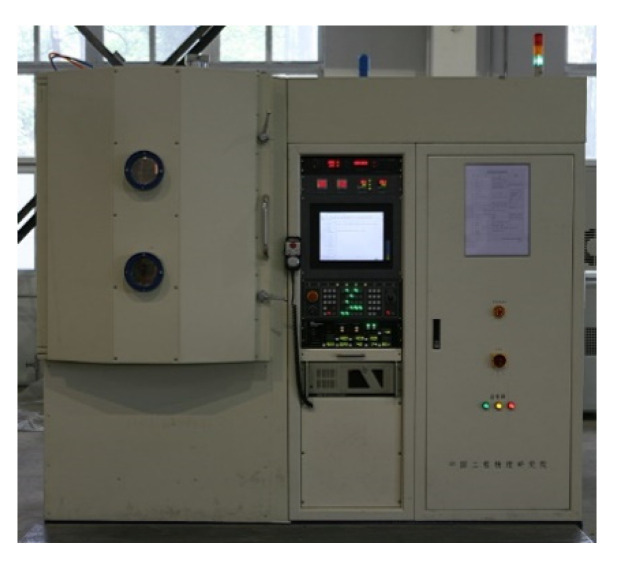
The self-developed IBF machine.

To validate the effectiveness of the proposed algorithm, repetitious experimental studies on flatness figuring was carried out based on the self-developed ion-beam figuring (IBF) machine. The experiment setup is shown in Figure 15. It can process planar, spherical and aspheric parts with a maximum size of 300 mm × 300 mm, and the positioning accuracy of the linear axis is below 0.005 mm. This machine can be used for the corresponding experimental verification of the dwell time solving algorithm. The specific parameters of the polishing processing are shown in Table 1.

**Figure 16:**
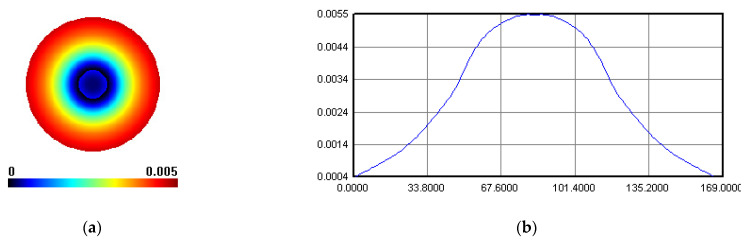
Removal function in case 1: (**a**) surface map, (**b**) surface profile.

IBF spots were taken on the IBF machine, and the results are shown in Figure 16. The polishing result was detected by the INF300H-LP-WM interferometer made in China, with RMS repeatability of less than 0.3 nm. The removal function result of IBF, shown in Figure 16, was tested on a fixed point, and polishing time was 60 s. The peak removal rate (PRR) of influence function was 0.30614 λ/min, where λ was 658 nm, and the volume removal rate (VRR) was 0.056474 mm^3^/min.

### 4.2. Results and Discussion

#### 4.2.1. Experiment Case 1

The workpiece used for figuring is silica flat and the diameter is φ120 mm. The scanning path is in raster with a step of 1.5 mm and spacing of 3.0 mm. Initial flatness was PV 0.259 λ, RMS 0.050 λ; Predicted flatness was PV 0.051 λ and RMS 0.004 λ, with a convergence rate of 80.0% and 92%, respectively. 

**Figure 17:**
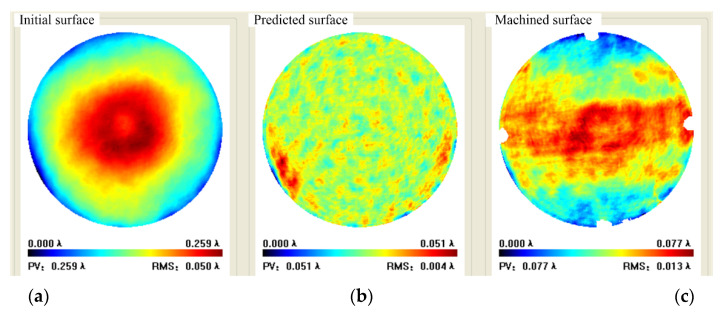
Full aperture data in case 1: (**a**) Initial surface, (**b**) Predicted surface, (**c**) Machined surface.

**Figure 18:**
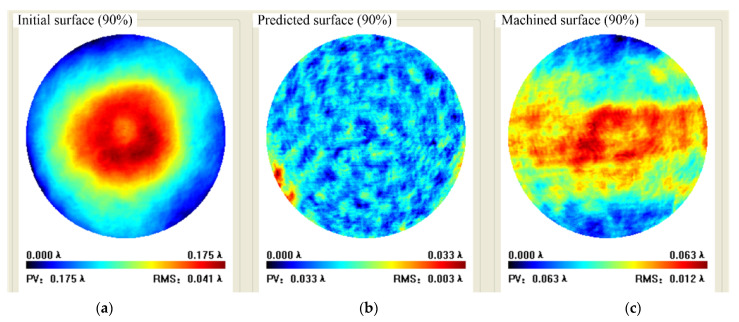
Case 1 90% aperture data: (**a**) Initial surface, (**b**) Predicted surface, (**c**) Machined surface.

Experimental result indicated that full-aperture machined flatness was PV 0.077 λ and RMS 0.013 λ with a convergence rate of 70.3% and 74.5% respectively, where the missing data in the periphery was caused by the fixture. The aperture machined flatness of 90% was PV 0.063 λ and RMS 0.012 λ with a convergence rate of 64% and 70% respectively, as shown in Figure 17 and Figure 18.

#### 4.2.2. Experiment Case 2

**Figure 19:**
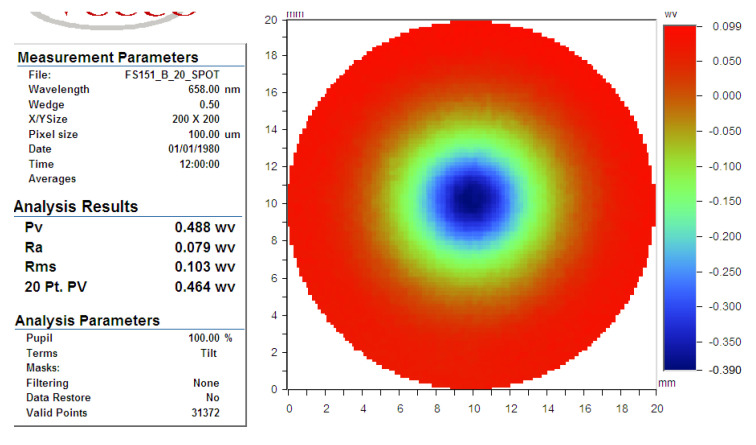
Removal function in case 2.

The universal measuring software was employed. The PRR of influence function was 0.244 λ/min, and the VRR was 0.0098 mm^3^/min as shown in Figure 19.

**Figure 20:**
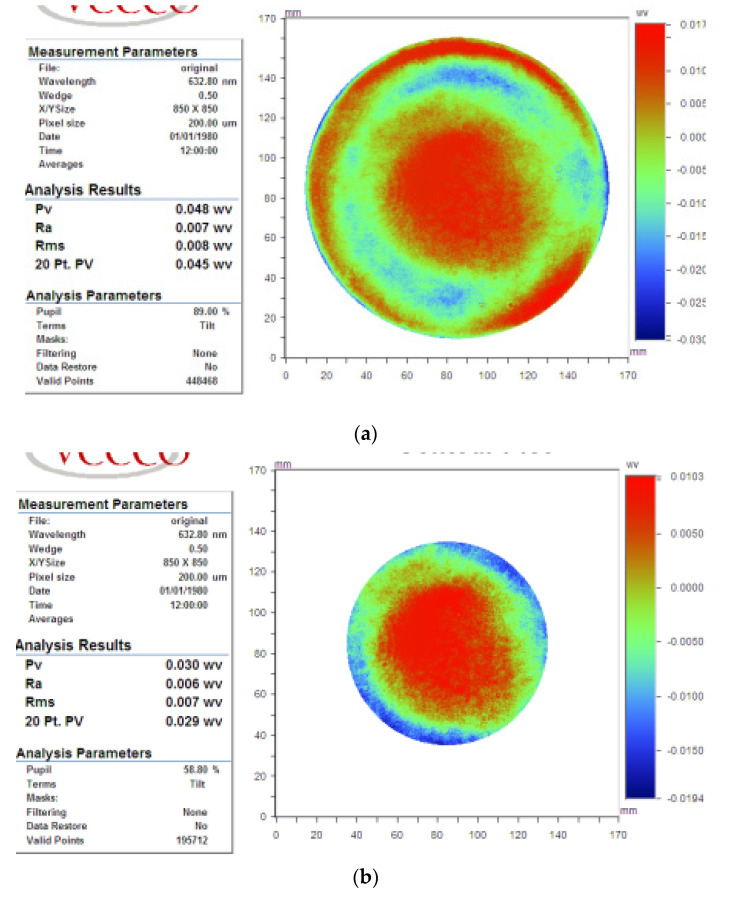
Initial surface in case 2: (**a**) φ150 mm aperture, (**b**) φ100 mm aperture.

The workpiece is fused silica with diameter of φ170 mm. Surface map is measured using the relative accuracy method. Initial flatness of φ150 mm aperture is PV_φ150_ = 0.048 λ, RMS_φ150_ = 0.008 λ, and those of φ100 mm aperture are PV_φ100_ = 0.030 λ, RMS_φ100_ = 0.007 λ respectively, as shown in Figure 20.

**Figure 21:**
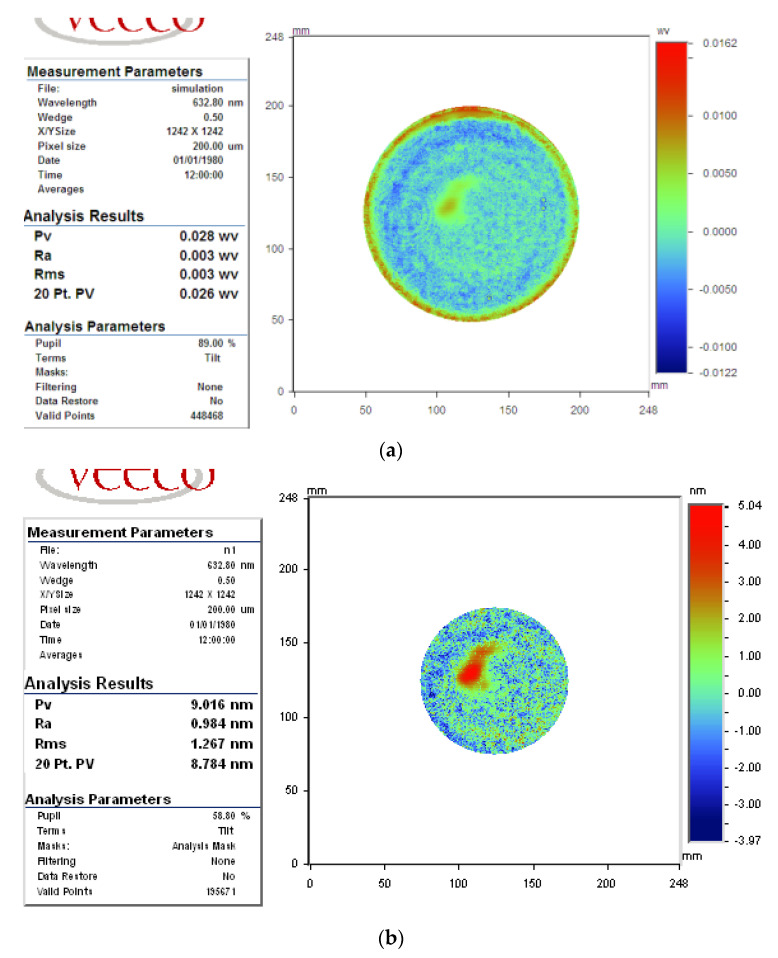
Predicted surface in case 2 (**a**) φ150 mm aperture (**b**) φ100 mm aperture.

The scanning path was rasterized with 2 mm spacing, and the predicted machine time was 83.8 min; the predicted flatness of φ150 mm aperture was PV_c150_ = 0.025 λ, RMS_c150_ = 0.003 λ; and those of φ100 mm were PV_c100_ = 0.014 λ; RMS_c100_ = 0.002 λ, as illustrated in Figure 21.

**Figure 22:**
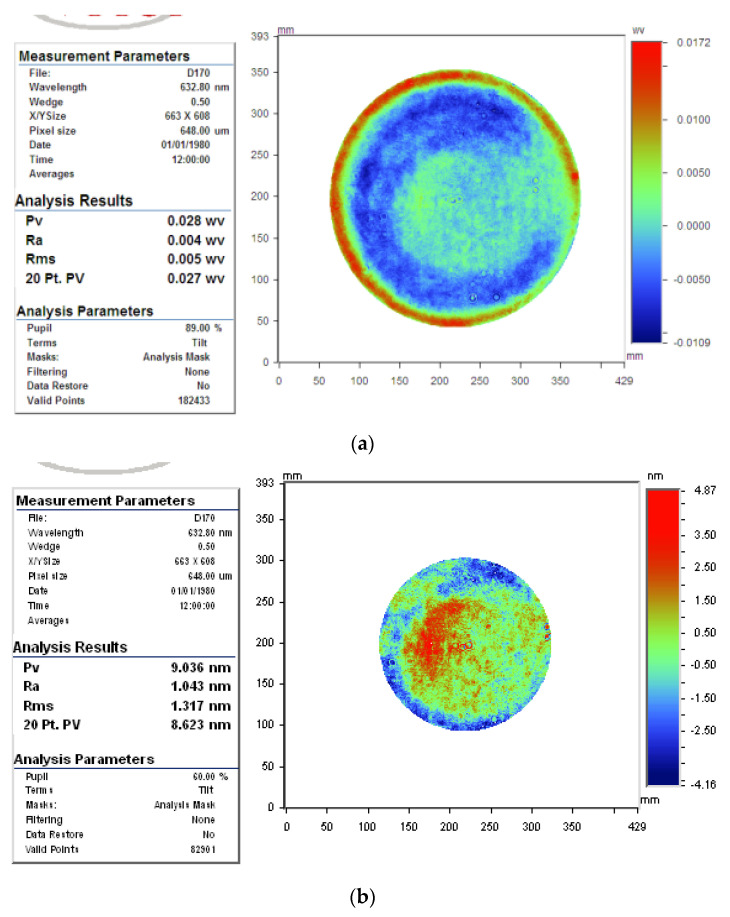
Polished surface in case 2: (**a**) φ150 mm aperture, (**b**) φ100 mm aperture.

The practical polishing time was 84 min. The surface map was also measured using the relative accuracy method. The polished flatness of the φ150 mm aperture was PV_r150_ = 0.028 λ, and RMS_r150_ = 0.005 λ, and those of φ100 mm were PV_cr100_ = 0.014 λ, and RMS_c100_ = 0.002 λ, as shown in Figure 22.

Simulated convergence rate was

(22) $$\eta_{c150}=\frac{pv_{o150}-pv_{c150}}{pv_{o150}}\times 100\%=47.9\%$$

And the practical convergence rate was

(23) $$\eta_{r150}=\frac{pv_{r150}-pv_{r150}}{pv_{r150}}\times 100\%=41.7\%$$

Taking the surface PV value of φ100 mm aperture as the evaluation index, the predicted convergence rate was 52.0%, and the practical convergence rate was 51.9%.

## 5. Conclusions

In this paper, an elementary approximation of the dwell time algorithm for single-peak rotational symmetry removal function was presented. The work showed that it is engineeringly acceptable to use the cone distribution as the removal function to approximate expressions of all the subaperture polishing with a single-peak rotational symmetry removal function, such as CCOS, IBF, or BP. The dwell time algorithm model and computation method were given. When the distance of node discretization was not more than the radius of the support domain of removal function, the coupling characteristic of the deconvolution problem could be eliminated by using the elementary approximation solution proposed in this paper. Theoretical analysis, numerical simulation and experimental results show that the proposed method had a lower calculated residual error than the initial value by one order of magnitude, and had a higher approximation ability. The flatness of φ150 mm and φ100 mm workpieces achieved PVr_150_ = 0.028 λ and PVcr_100_ = 0.014 λ, respectively. 

In contrast to conventional dwell time algorithms, this work transformed the superposition and coupling features of the deconvolution problem into a simple calculation of the discretization function value. When the discrete nodes were doubled, the time weight of each node was then halved; consequently, the total time remained unchanged. The approximation ability or smoothness of the deconvolution result was greatly increased, which agreed with the engineering reality that total polishing time remains unchanged if the total removal amount and removal function are unchanged. Compared with conventional methods, the proposed algorithm has obvious advantages for improving calculation efficiency and smoothness, which is of great significance for the efficient computation of large-aperture optical polishing. 

Nevertheless, the calculation accuracy of the proposed dwell time algorithm is related to the symmetry of the removal function and its approximation error. Meanwhile, the dwell time algorithm has a limited ability to correct mid-to-high frequency errors determined by the sampling characteristics of the discretization nodes and the scale of the removal function. The approximation error in the edge area of the workpiece needs further analysis.

## Figures and Tables

**Table 1:** Parameters of the polishing process.

Parameter	Value
Ion beam voltage	800 V
Ion beam current	60 mA
Ion beam Angle	0°
Processing distance	150 mm
Grating spacing	2 mm

## Data Availability

NOMENCLATURE is contained within the main text.

## Nomenclature

R(x, y)	Removal function
D(x, y)	Dwell time function
H (Xi, Yj)	Target removal amount
A	Peak value of the removal function center
fabs	Function of taking absolute value
t(xi, yi)	Dwell time at the i th path node
h(xk, yk)	Desired amount of removed material at the k th figure-control node
Nt	Total number of the path nodes
Nh	Total number of the figure-control nodes
r(xk-xi, yk-yi)	Amount of removed material at the k th figure-control node
L	Discretization distance of nodes
CCOS	Computer controlled optical surfacing
MRF	Magnetorheological finishing
IBF	Ion beam figuring
BP	Bonnet polishing
